# Microstructure reconstruction of 2D/3D random materials via diffusion-based deep generative models

**DOI:** 10.1038/s41598-024-54861-9

**Published:** 2024-02-29

**Authors:** Xianrui Lyu, Xiaodan Ren

**Affiliations:** College of Civil Engineering, Tongji University, Shanghai, 200092 People’s Republic of China

**Keywords:** Composites, Computer science, Composites, Computer science

## Abstract

Microstructure reconstruction serves as a crucial foundation for establishing process–structure–property (PSP) relationship in material design. Confronting the limitations of variational autoencoder and generative adversarial network within generative models, this study adopted the denoising diffusion probabilistic model (DDPM) to learn the probability distribution of high-dimensional raw data and successfully reconstructed the microstructures of various composite materials, such as inclusion materials, spinodal decomposition materials, chessboard materials, fractal noise materials, and so on. The quality of generated microstructure was evaluated using quantitative measures like spatial correlation functions and Fourier descriptor. On this basis, this study also achieved the regulation of microstructure randomness and the generation of gradient materials through continuous interpolation in latent space using denoising diffusion implicit model (DDIM). Furthermore, the two-dimensional microstructure reconstruction was extended to three-dimensional framework and integrated permeability as a feature encoding embedding. This enables the conditional generation of three-dimensional microstructures for random porous materials within a defined permeability range. The permeabilities of these generated microstructures were further validated through the application of the lattice Boltzmann method. The above methods provide new ideas and references for material reverse design.

## Introduction

The geometric morphologies of engineering materials play a pivotal role in elucidating their performance characteristics, such as the light-capturing efficiency in silicon solar cells^1^, mass transport in porous materials^2^ and the electrical properties of polymer nanocomposite^3^. Consequently, in the modern materials science, there has been a notable shift away from traditional trial-and-error methods for material discovery. Instead, there is a growing emphasis on material inverse design, focusing on the elucidation of the intricate relationships between process, structure, and property (PSP)^4^. The pursuit of identifying the mapping between PSP enables targeted microstructure design to guide the development of novel materials with desired performance characteristics, which has become a mainstream research framework in the field of material development.

However, the establishment of a comprehensive mapping between microstructures and material properties necessitates the acquisition of extensive microstructural data for high-throughput analysis. The significant experimental costs required to obtain detailed microstructural information have prompted scholars to extensively explore microstructure characterization and reconstruction (MCR) technologies^5^, including the two-point correlation function, lineal-path function, and two-point clustering correlation function, among others, as well as the spectral density function^6–8^. In fact, microstructural reconstruction methods based on the aforementioned statistical descriptors, such as Yeong–Torquato (YT) algorithm^9^ and parameterized random field, are essentially low-order statistical information representations, rather than a complete description of the distribution of the original data. Furthermore, it has been noted by some scholars^10^ that in certain heterogeneous materials, discrepancies in morphology and macroscopic properties may arise even when the microstructures share identical low-order statistical information. Although efforts to refine the YT algorithm’s energy function^11–13^ aim to improve the representation of statistical information, it should be acknowledged that the YT method fundamentally operates as an iterative optimization algorithm. The computational cost of this method increases with the complexity of the energy function, and the quality of the reconstruction depends on the care choice of the characterization function. In light of these considerations, the development of a fast and efficient reconstruction method that incorporates high-order statistical information and is versatile enough to be applied across different material systems remains a critical research objective.

In essence, the pixel (2D) or voxel (3D) representation of microstructures can be conceptualized as a complex probability distribution governed by ultra-high-dimensional random variables. Meanwhile, a fundamental objective of deep learning is to learn the manifold structures and probability distributions within data^14,15^, and inherently possesses a formidable capability to manipulate such high-dimensional probability distributions. This capability has spurred increasing interest in applying deep learning to articulate the high-dimensional probability distributions characterizing microstructures^16–18^, with a particular focus on probability density estimation and the mapping between manifolds via neural networks’ nonlinear mapping capacities. This endeavor is primarily categorized into explicit and implicit probability density estimation methods^19^. Explicit probability density estimation models, such as PixelRNN or PixelCNN, employ the chain rule of probability to reframe image generation as a sequence generation task, representing it as a product of joint conditional probabilities. While successful applications of reconstructing microstructures have been achieved^20^, it requires a predetermined order for pixel generation, and the generation speed is often excessively slow. Conversely, the approximate explicit probability estimation model-variational autoencoder (VAE)^21^ and the implicit probability density estimation model-generative adversarial network (GAN)^22^ are widely used in the field of microstructure reconstruction in the past decade. VAE transforms the challenge of probability density estimation into function approximation. It achieves this by employing maximum likelihood estimation to bring a mixed Gaussian distribution closer to the true underlying distribution. This approach has enabled the successful reconstruction of varied materials such as sandstone and metamaterials^23,24^. Additionally, Xu et al.^25^ developed a deep learning model based on VAE for reconstructing deterministic and stochastic material microstructures, achieving not only randomness control but also the introduction of  reliable quantitative metrics for randomness assessment. However, VAEs are not without their challenges, notably the “maximum likelihood training paradigm”, which can result in the generation of blurry images^26^. In contrast, GANs achieve Nash Equilibrium between generator and discriminator through adversarial training, yielding superior generative performance. Many scholars^16,27–29^ have proposed various microstructure reconstruction algorithms based on GANs and applied in polycrystalline grains, perovskite, carbon fiber rods, rocks and so on. Nevertheless, the training of GAN is unstable due to the adversarial nature of the loss functions^30,31^. Additionally, GANs are vulnerable to mode collapse, a situation in which they repetitively generate the same image. These limitations also impede the further practical application of GANs.

Recently, the diffusion model has emerged as the frontrunner in the field of AI-generated content (AIGC), surpassing both VAE and GAN. The most advanced text-to-image model, such as OpenAI’s DALL$\cdot$E 2 and Google’s Imagen, are built upon the diffusion model. The inspiration behind the diffusion model stems from non-equilibrium thermodynamics. In contrast to GANs, the diffusion model operates without the need for adversarial training, bringing with it added advantages in terms of training efficiency, scalability, and parallelism. In terms of generative capabilities, in addition to achieving state-of-the-art image quality, diffusion models exhibit a robust ability to encompass various patterns and generate diverse outputs. Beyond image generation, diffusion models have demonstrated significant potential in a wide array of fields, including computer vision^32–35^, natural language processing^36,37^ , time series modeling^38,39^, multimodal modeling^40,41^, and more.

Encouragingly, diffusion models have also shone brightly in the fields of material synthesis and structural reconstruction. However, it mainly focuses on biomaterials or medical imaging^42,43^, such as protein modeling and cell shape prediction^44–46^. There are only a few studies dedicated to examining the reconstruction of the microstructures of composite materials. In the limited existing literature, some scholars^47–49^ successfully reconstructed the microstructure of polycrystalline alloys, carbonates, ceramics, fiber composites, and other materials based on diffusion model. Yet, these investigations predominantly target two-dimensional microstructures, with limited examination of the morphological characteristics and the latent space of the diffusion model. In another study, Nikolaos and Sun’s work^50^ represents a pioneering effort to generate microstructures aligned with specific performance targets using context feature vectors within the mechanical MNIST dataset. However, this research is constrained by the dataset’s low resolution and simple microstructures, leaving a gap in the evaluation of more complex and higher-resolution random microstructures. Furthermore, Vlassis et al.^51^ utilizes VAE to reduce the dimensionality of 3D point cloud structures to a low dimensional latent space, and reconstructs the 3D structure of sand particles after training the diffusion model in the latent space. Nevertheless, this approach only generates individual particles and lacks the incorporation of multi-scale features in microstructure generation.

Therefore, this study proposes an end-to-end microstructure reconstruction method based on data-driven denoising diffusion probabilistic diffusion model (DDPM) for heterogeneous engineering materials in two and three dimensions. Initially, the microstructure datasets were established for various composite materials, including regular inclusions, chessboard structures, spinodal decomposition materials, and random materials. The above microstructures with resolutions of $64 \times 64$ and $128 \times 128$ were reconstructed successfully by DDPM, and the statistical descriptors such as two-point correlation function and lineal-path function were used to evaluate the quality of generated microstructures. Meanwhile, Fourier descriptor also was used to verify the morphological similarity between the both. On this basis, this study fully explored and utilized the latent space of diffusion models through the deterministic generation of denoising diffusion implicit model (DDIM), achieving the regulation of the randomness of generated microstructures. Following this, the study extended the two-dimensional DDPM reconstruction method to encompass three-dimensional conditional generation, which was verified to generate three-dimensional random porous materials with a specific range of permeability.

## Methods

### Microstructure reconstruction via DDPM

**Figure 1:**
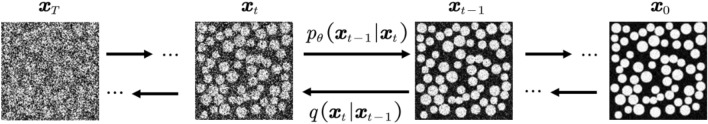
The forward noising process and reverse denoising process in diffusion model.

The DDPM comprises two primary components: the forward diffusion process and the reverse denoising process. In the forward diffusion process, Gaussian noise is incrementally introduced to the original image tensor, ultimately transforming it into a noise image conforming to a standard normal distribution. Conversely, the reverse denoising process progressively eliminates noise from a given Gaussian noise image, ultimately restoring the original image from its noisy counterpart. These two processes are shown in Fig.  1.

In contrast to the single-step mixed Gaussian distribution to approximate the original data distribution in VAE, the diffusion model employs a normal distribution to approximate incremental changes at each step. This approach enables the diffusion model to overcome the limitations typically associated with the fitting capacity of traditional single-step VAE.

Specifically, in the forward process, Gaussian noise $\varepsilon$ is continuously added to the given initial data distribution ${\textbf{x}}_0 \sim q({\textbf{x}})$, and the variance sequence of noise is $\beta _t$, which gradually increases with time steps. Each additional step of noise generates a new latent variable ${\textbf{x}}_t$ with a distribution of $q\left( {\textbf{x}}_{{\textbf{t}}}\left| {\textbf{x}}_{{\textbf{t}}-1} \right. \right)$.

1 $$\begin{aligned} q\left( {\textbf{x}}_t|{\textbf{x}}_{t-1} \right) ={\mathcal {N}}\left( {\textbf{x}}_t;\sqrt{1-\beta _t}{\textbf{x}}_{t-1},\beta _t{\textbf{I}} \right) \quad q\left( {\textbf{x}}_{1:T}|{\textbf{x}}_0 \right) =\prod \limits _{t=1}^T{q}\left( {\textbf{x}}_t|{\textbf{x}}_{t-1} \right) . \end{aligned}$$

Based on the Markov chains hypothesis, as *t* progresses, the final data distribution ${\textbf{x}}_T$ converges towards an anisotropic independent Gaussian distribution and can be directly derived from ${\textbf{x}}_0$ and $\beta _t$,

2 $$\begin{aligned} q\left( {\textbf{x}}_t|{\textbf{x}}_0 \right) ={\mathcal {N}}\left( {\textbf{x}}_t;\sqrt{{\bar{\alpha }}_t}{\textbf{x}}_0,\left( 1-{\bar{\alpha }}_t \right) {\textbf{I}} \right) , \end{aligned}$$

where $$\alpha _t:=1-\beta _t$$ and $${\bar{\alpha }}_t:=\prod _{i=0}^t{\alpha _i}$$.

In the reverse process, it can be demonstrated that $q\left( {\textbf{x}}_{t-1}|{\textbf{x}}_t \right)$ also converges to a Gaussian distribution^52^. Consequently, a parameterized distribution $p_{\theta }\left( {\textbf{x}}_{{\textbf{t}}-1}\left| {\textbf{x}}_{{\textbf{t}}} \right. \right)$ is established in the reverse process, thereby,

3 $$\begin{aligned} \quad p_{\theta }\left( {\textbf{x}}_{t-1}|{\textbf{x}}_t \right) ={\mathcal {N}}\left( {\textbf{x}}_{t-1};{\tilde{\mu }}_{\theta }\left( {\textbf{x}}_t,t \right) ,{\tilde{\beta }}_t\left( {\textbf{x}}_t,t \right) \right) . \end{aligned}$$

Combining the Bayesian formula with the forward diffusion Eq. (2), the posterior diffusion conditional probability can be explicitly expressed^53^ and the mean and variance are,

4 $$\begin{aligned}{} & {} {\tilde{\mu }}_{\theta }\left( {\textbf{x}}_t,{\textbf{x}}_0 \right) =\frac{1}{\sqrt{\alpha _t}}\left( {\textbf{x}}_t-\frac{1-\alpha _t}{\sqrt{1-{\bar{\alpha }}_t}}\varepsilon _{\theta } \right) , \end{aligned}$$

5 $$\begin{aligned}{} & {} {\tilde{\beta }}_t=\frac{1-{\bar{\alpha }}_{t-1}}{1-{\bar{\alpha }}_t}\cdot \beta _t. \end{aligned}$$

Consistent with the fundamental objective of generative model, it is crucial to minimize the Kullback–Leibler (KL) divergence between the parameterized probability distribution and the probability distribution of real images as effectively as possible. However, for the diffusion model, which operates across T time steps, it is necessary to extend the Evidence Lower Bound (ELBO) as used in VAE into a chain representation, which can be expressed as follows:

6 $$\begin{aligned} {\mathcal {L}}_{VLB}&={\mathbb {E}}_{q(x_{0:T})}\bigg [\log \frac{q(x_{1:T}\mid x_{0})}{p_{\theta }(x_{0:T})}\bigg ] \\&={\mathbb {E}}_q\left[ \underbrace{D_\text{KL}\left( q({\textbf{x}}_T|{\textbf{x}}_0)\parallel p_\theta ({\textbf{x}}_T)\right) }_{L_T}+\sum _{t=2}^T\underbrace{D_\text{KL}(q({\textbf{x}}_{t-1}|{\textbf{x}}_t,{\textbf{x}}_0)\parallel p_\theta ({\textbf{x}}_{t-1}|{\textbf{x}}_t)}_{L_{t-1}}-\underbrace{\log p_\theta ({\textbf{x}}_0|{\textbf{x}}_1)}_{L_0}\right] . \end{aligned}$$

The final loss function can be simplified as,

7 $$\begin{aligned} L_{t}^{\text {simple}}={\mathbb {E}}_{x_0,\epsilon _t}\left[ \Vert \epsilon _t-\epsilon _{\theta }\left( \sqrt{{\bar{\alpha }}_t}x_0+\sqrt{1-{\bar{\alpha }}_t}\epsilon _t,t \right) \Vert ^2 \right] . \end{aligned}$$

**Figure 2:**
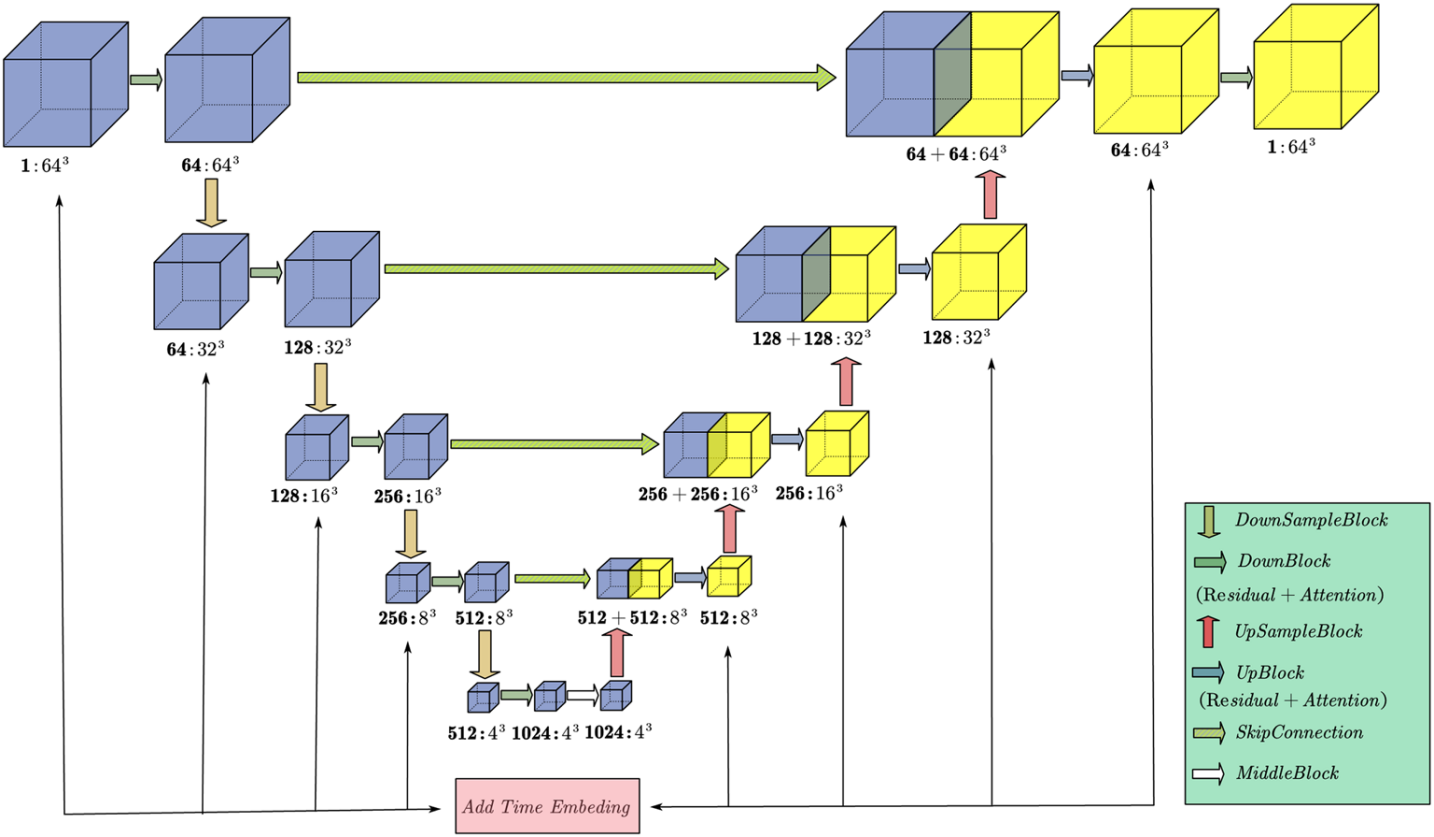
3D U-Net network architecture in diffusion model.

In DDPM, the noise corresponding to the time step is predicted by the U-Net and the topology of network designed in this study is shown in the Fig.  2. The model in this study underwent 500 training iterations on computer with NVIDIA GeForce RTX 4070 Ti and was optimized using the Adam optimizer.

**Figure 3:**
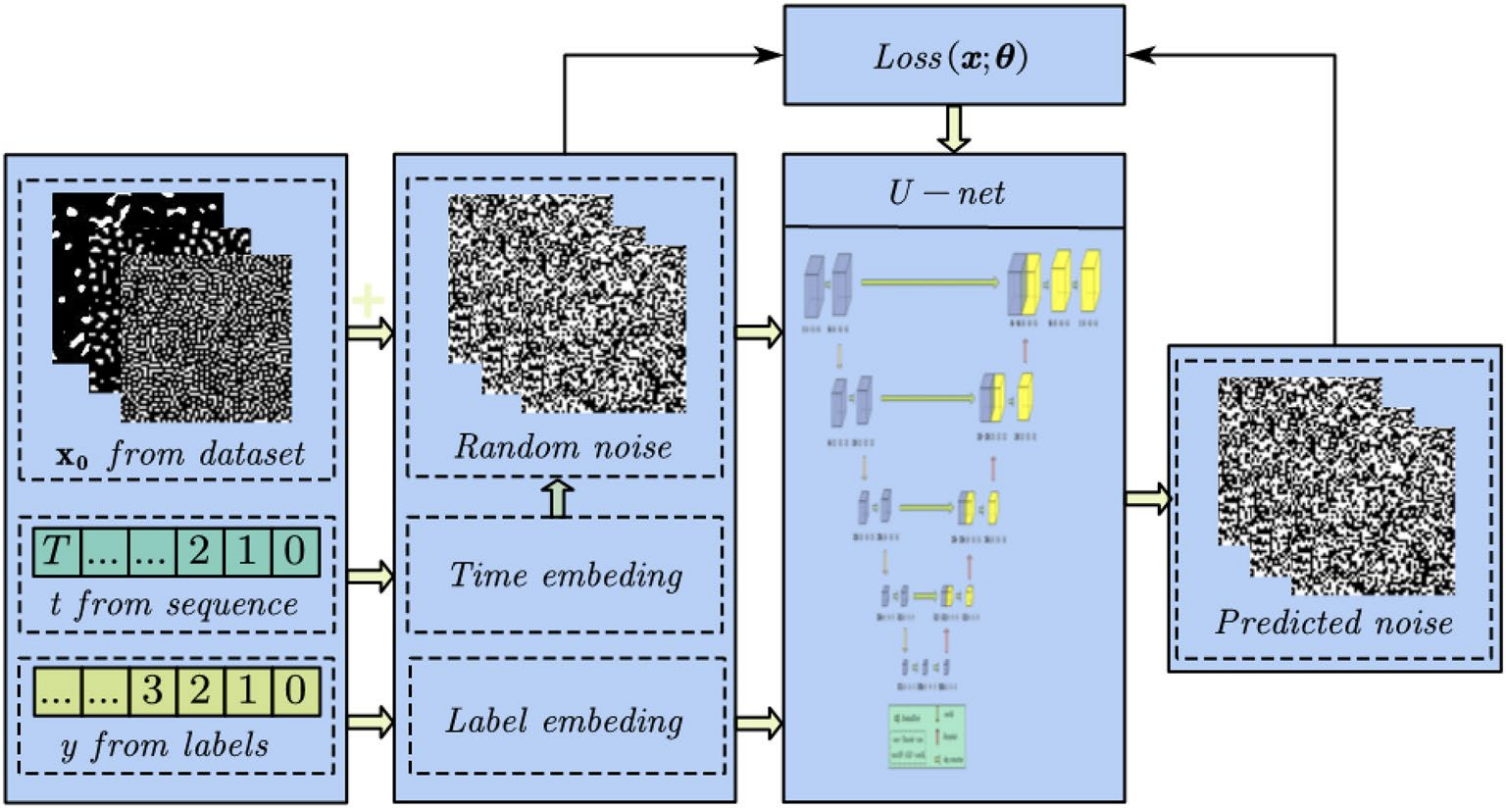
The training process of conditional diffusion model.

Meanwhile, in order to avoid additional training and adversarial attack effects in classifier guidance, in this study, we adopt the classifier-free guidance scheme. Specifically, this study incorporated supplementary feature encoding embeddings with time feature encoding into the diffusion model, enabling control over various physical attributes. The training diagram of the conditional diffusion model is shown in Fig.  3. The loss function of the conditional generation model can be expressed as,

8 $$\begin{aligned} {\mathbb {E}}_{{\varvec{x}}_0,{\varvec{y}}\sim {\tilde{p}}\left( {\varvec{x}}_0,{\varvec{y}} \right) ,\varvec{\varepsilon }\sim {\mathcal {N}}\left( 0,{\varvec{I}} \right) }\left[ \Vert \varvec{\varepsilon }-\varvec{\epsilon }_{\varvec{\theta }}\left( \sqrt{{\bar{\alpha }}_t}{\varvec{x}}_0+\sqrt{1-{\bar{\alpha }}_t}\varvec{\varepsilon _t}, {\varvec{y}}, t \right) \Vert ^2 \right] . \end{aligned}$$

Here, ${\varvec{y}}$ represents the feature embedding corresponding to the physical attribute, effectively serving as the label for microscopic physical attributes, such as elastic modulus or permeability.

### Deterministic reconstruction via DDIM

In DDPM, both forward and backward process are defined as a Markov chain. However, in the derivation of reverse diffusion process, only the edge distribution $q\left( {\textbf{x}}_t\left| {\textbf{x}}_0 \right. \right)$ is required. This implies that the inference distribution in the reverse process is not necessarily required to follow Markov chains. Therefore, in the DDIM, the distribution of reverse process is directly defined as^54^,

9 $$\begin{aligned} q_{\sigma }\left( {\textbf{x}}_{t-1}|{\textbf{x}}_t,{\textbf{x}}_0 \right) ={\mathcal {N}}\left( {\textbf{x}}_{t-1};\sqrt{{\bar{\alpha }}_{t-1}}{\textbf{x}}_0+\sqrt{1-{\bar{\alpha }}_{t-1}-\sigma _{t}^{2}}\frac{{\textbf{x}}_t-\sqrt{{\bar{\alpha }}_t}{\textbf{x}}_0}{\sqrt{1-{\bar{\alpha }}_t}},\sigma _{t}^{2}{\textbf{I}} \right) . \end{aligned}$$

By eliminating the Markov chain hypothesis in the reverse process, during the sampling, a larger step can be set to accelerate the generation process. According to Eq. (9), the following equation can be employed to facilitate sampling from ${\textbf{x}}_t$ to ${\textbf{x}}_{t-1}$ during the reverse diffusion process.

10 $$\begin{aligned} {\textbf{x}}_{t-1}=\sqrt{\alpha _{t-1}}\underbrace{\left( \frac{{\textbf{x}}_t-\sqrt{1-\alpha _t}\epsilon _{\theta }({\textbf{x}}_t,t)}{\sqrt{\alpha _t}}\right) }_{\text {predicted }\mathbf {x_0}}+\underbrace{\sqrt{1-\alpha _{t-1}-\sigma _t^2}\cdot \epsilon _{\theta }({\textbf{x}}_t,t)}_{\text {direction pointing }to\quad \mathbf {x_t}}+\underbrace{\sigma _t\epsilon _t}_{\text {random}}. \end{aligned}$$

The equation above decomposes the generation process into three components: the first is directly predicted ${\textbf{x}}_0$ by ${\textbf{x}}_t$, the second is the part pointing towards ${\textbf{x}}_t$, and the third entails random noise $\epsilon _t$. The unknown parameter $\sigma _t$ is defined as follows:

11 $$\begin{aligned} \sigma _{t}^{2}=\eta \cdot {\tilde{\beta }}_t=\eta \cdot \sqrt{\left( 1-\alpha _{t-1} \right) /\left( 1-\alpha _t \right) }\sqrt{\left( 1-\alpha _t/\alpha _{t-1} \right) }. \end{aligned}$$

When $\eta =1$, the variance setting in DDIM aligns with that in DDPM. Conversely, when $\eta =0$, random noise is absent, resulting in deterministic sampling. Considering that there is a continuity mapping between the latent space and the real data in DDIM, continuous sampling in the latent space can induce the dynamic evolution continuously of the real microstructures. This evolution also forms the foundation for subsequent regulation of randomness and the generation of gradient materials.

## Results

### Dataset generation

To assess the efficacy of diffusion models in microstructure reconstruction with varying levels of complexity and randomness, this study initially established a database comprising various types of structural features. These included fiber inclusion microstructure, circular inclusion microstructure, texture microstructure, random inclusion microstructure, spinodal decomposition microstructure, Voronoi microstructure, fractal noise microstructure, and chessboard microstructure. It is worth noting that these structural features share consistent characteristics with the microstructures of real materials^25^, such as Voronoi structures in polycrystalline materials^55^, quasi random and texture structural features in electrodes^56,57^, and characteristics of circular inclusions in ${\text{NaYF}}_{4}$ materials^58^. In the described database, the scale of each microstructure in the above database is 1000. Except for the image size of circular inclusion microstructure and fractal noise microstructure, which is $128 \times 128$, the size of all other microstructures is $64 \times 64$.

Various reconstruction algorithms were employed to generate database. These algorithms encompassed random field method based on random harmonic functions^59^, reconstruction methods grounded in physical descriptors and material image analysis tool-PoreSpy^60^. The specific methods for generating databases can be found in Supplementary. In addition, databases of metamaterials and three-dimensional porous materials were obtained from open access structure libraries^2,17,61,62^.

### Qualitative and quantitative comparison of real and reconstructed microstructures

**Figure 4:**
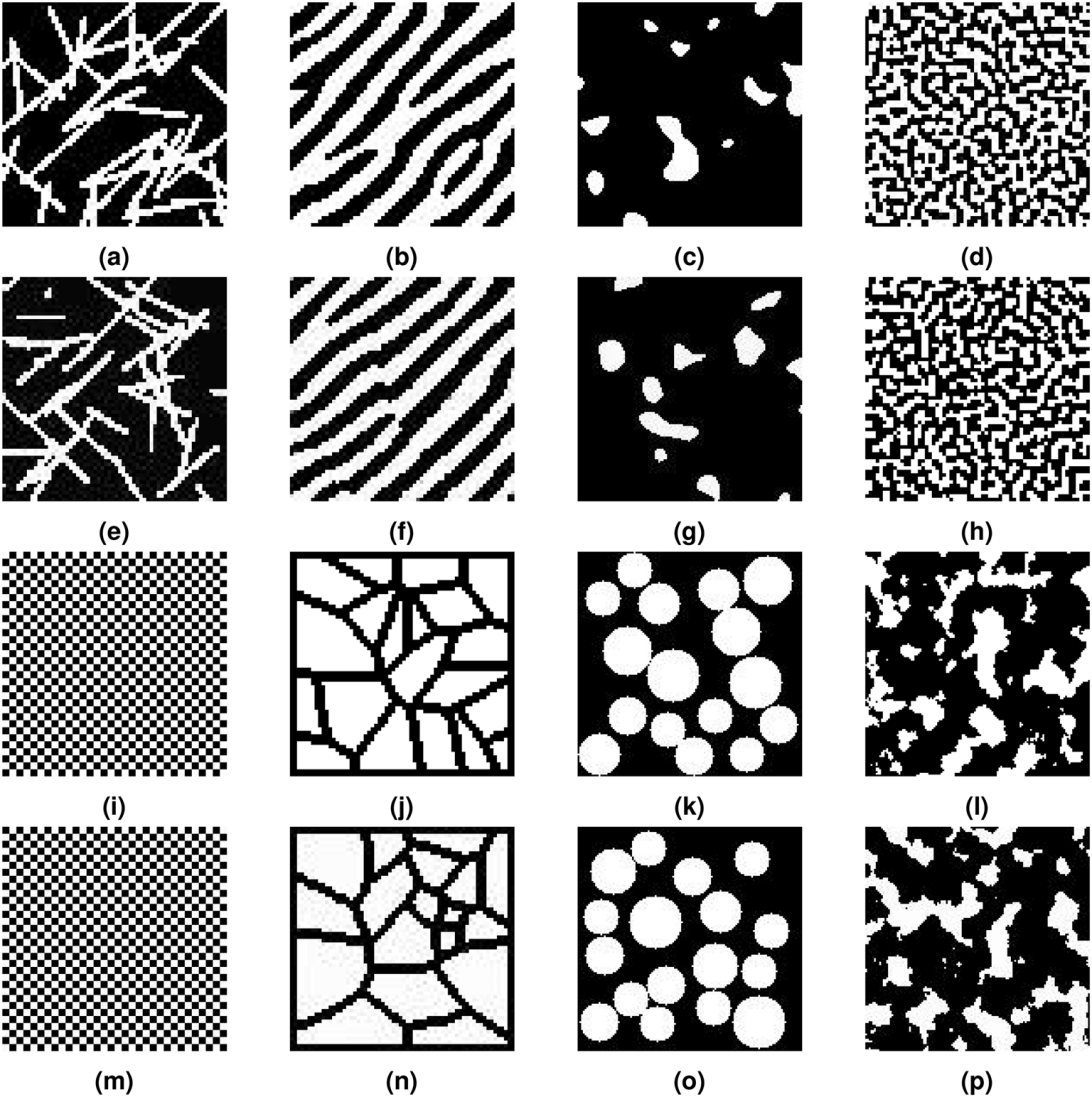
Comparison between generated microstructures based on DDPM and original microstructures;(**a–d,i–l**) Original microstructures. (**e–h,m–p**) Generated microstructures.

In this section, the microstructures of random fiber inclusion materials, texture materials, random materials, spinodal decomposition materials, chessboard structures, Voronoi structured materials, circular inclusion and fractal noise materials were successfully reconstructed based on DDPM. Figure  4 provides a comparison between the original microstructure and the reconstructed microstructure, revealing no noticeable visual distinctions.

**Figure 5:**
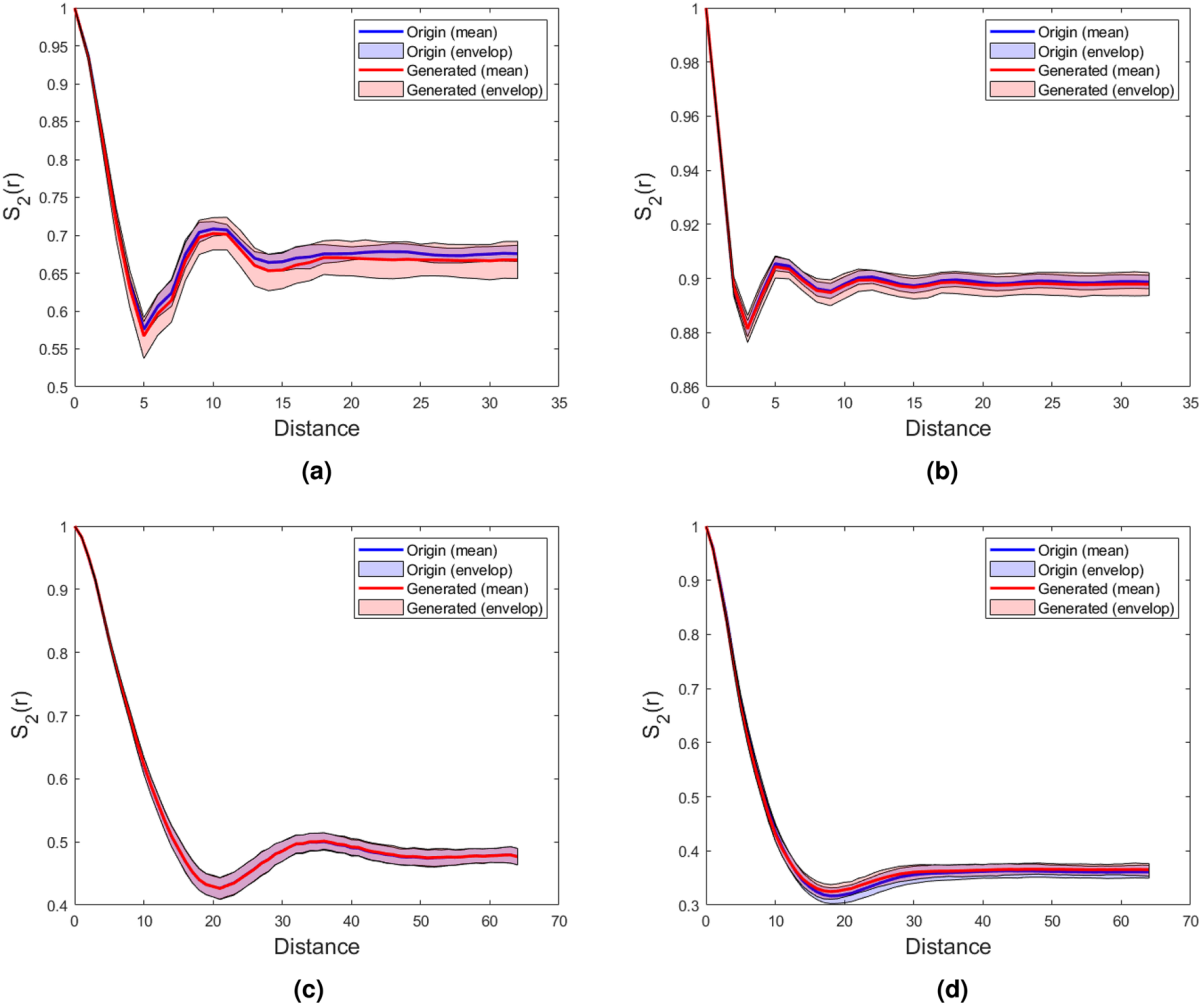
Comparison of $S_2 \left( r \right)$ between generated microstructures and original microstructures; (**a**) Texture material; (**b**) Spinodal decomposition. (**c**) Circular inclusion, (**d**) Fractal noise.

**Figure 6:**
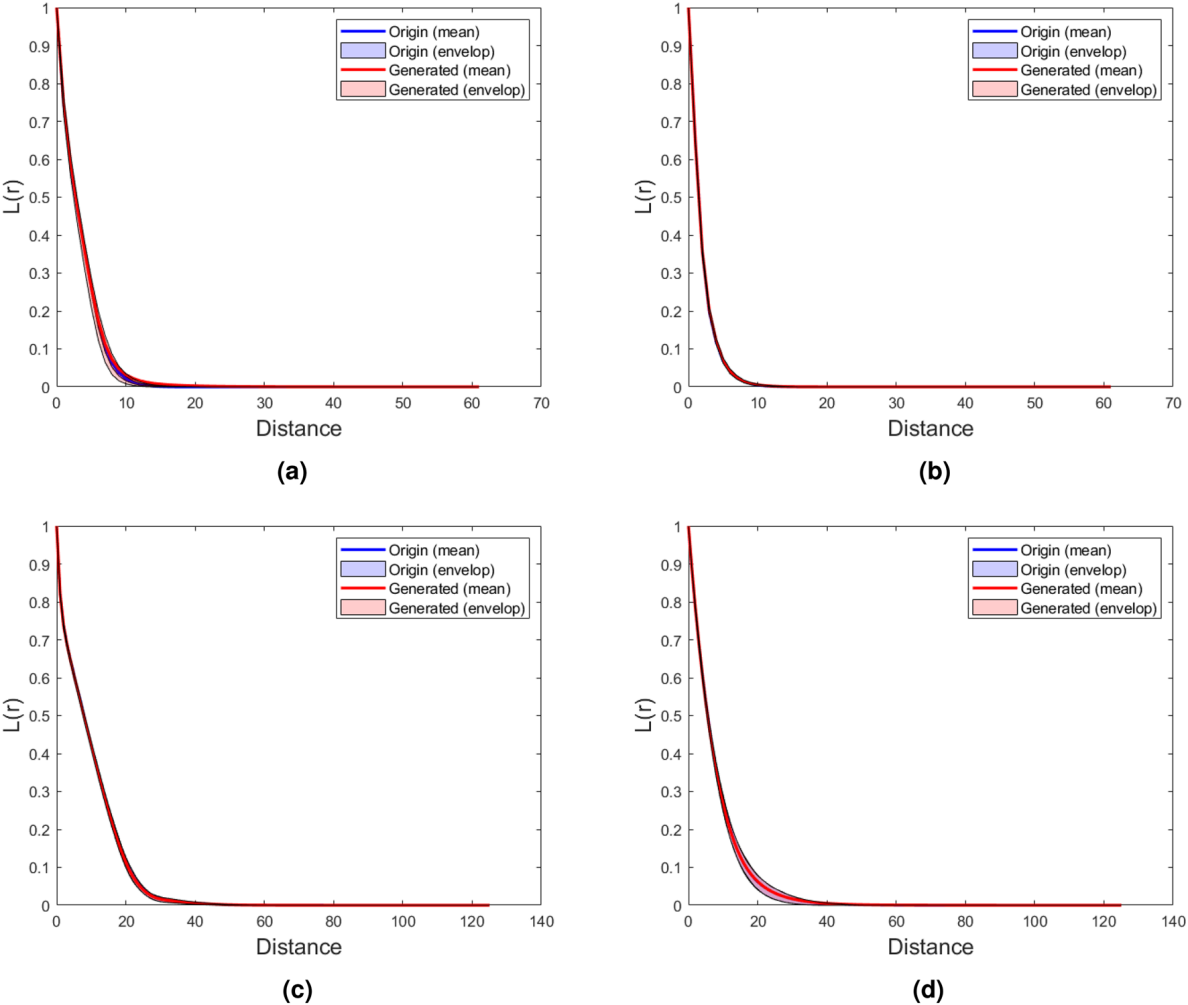
Comparison of $L\left( r\right)$ between generated microstructures and original microstructures; (**a**) Texture material. (**b**) Spinodal decomposition. (**c**) Circular inclusion. (**d**) Fractal noise.

Following this, the spatial correlation functions, including the two-point correlation function $S_2\left( r \right)$^8,63^ and lineal-path function $L\left( r\right)$^64^, were computed to quantitatively assess the microstructure’s morphology, as illustrated in the Figs.  5 and 6. The comparison clearly demonstrates that the two-point correlation function and lineal-path function of both low and high-resolution microstructures reconstructed using DDPM exhibit a remarkable degree of consistency with the original microstructures.

**Figure 7:**
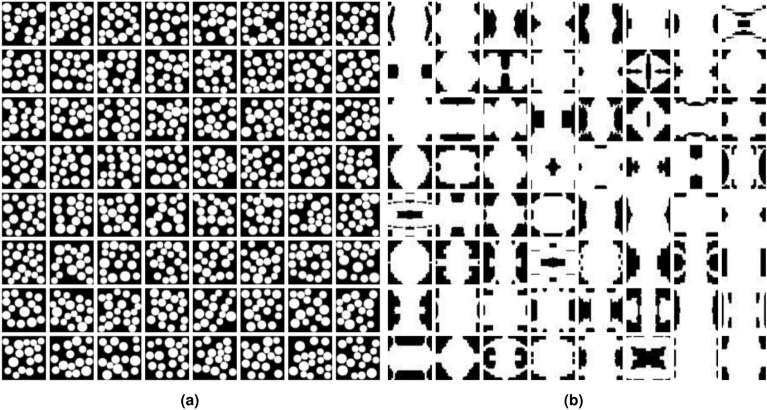
Diversified generated microstructures based on DDPM; (**a**) Circular inclusions. (**b**) Metamaterials.

To verify the diversity of model generation ability, random sampling reconstruction was performed on circular inclusions and metamaterials , as shown in Fig.  7. On the other hand, this also suggests that DDPM has the capability to prevent the emergence of mode collapse problems.

In addition to quantitatively comparing the consistency of statistical information between the original random microstructure and the generated random microstructure, this study also employs Fourier descriptor^65,66^ to compare the morphological similarity between the two. Within the microstructure analysis, phase boundaries can be precisely delineated as sequences of coordinates $s\left( k\right) =\left[ x\left( k\right) ,y\left( k\right) \right]$, $k=0,1,2...K-1$, where each pair represents a point along the boundary. These coordinates are subsequently transformed into complex numbers, $s\left( k \right) =x\left( k \right) +jy\left( k \right)$. A Discrete Fourier Transform (DFT) is then applied to this one-dimensional sequence $s\left( k\right)$, resulting in the Fourier descriptors of the boundary $a\left( u\right)$,

12 $$\begin{aligned} a\left( u \right) =\sum _{k=0}^{K-1}{s}\left( k \right) \text {e}^{-j2\pi uk/K}, \end{aligned}$$

where $u=0,1,2...K-1$. Here, the shape information of the boundary is concisely encoded in the feature vectors of the Fourier descriptor. Moreover, a simple normalization procedure applied to the Fourier descriptors ensures their invariance to translation, rotation, and scaling, enhancing their utility in comparative analyses across different microstructural configurations.

**Figure 8:**
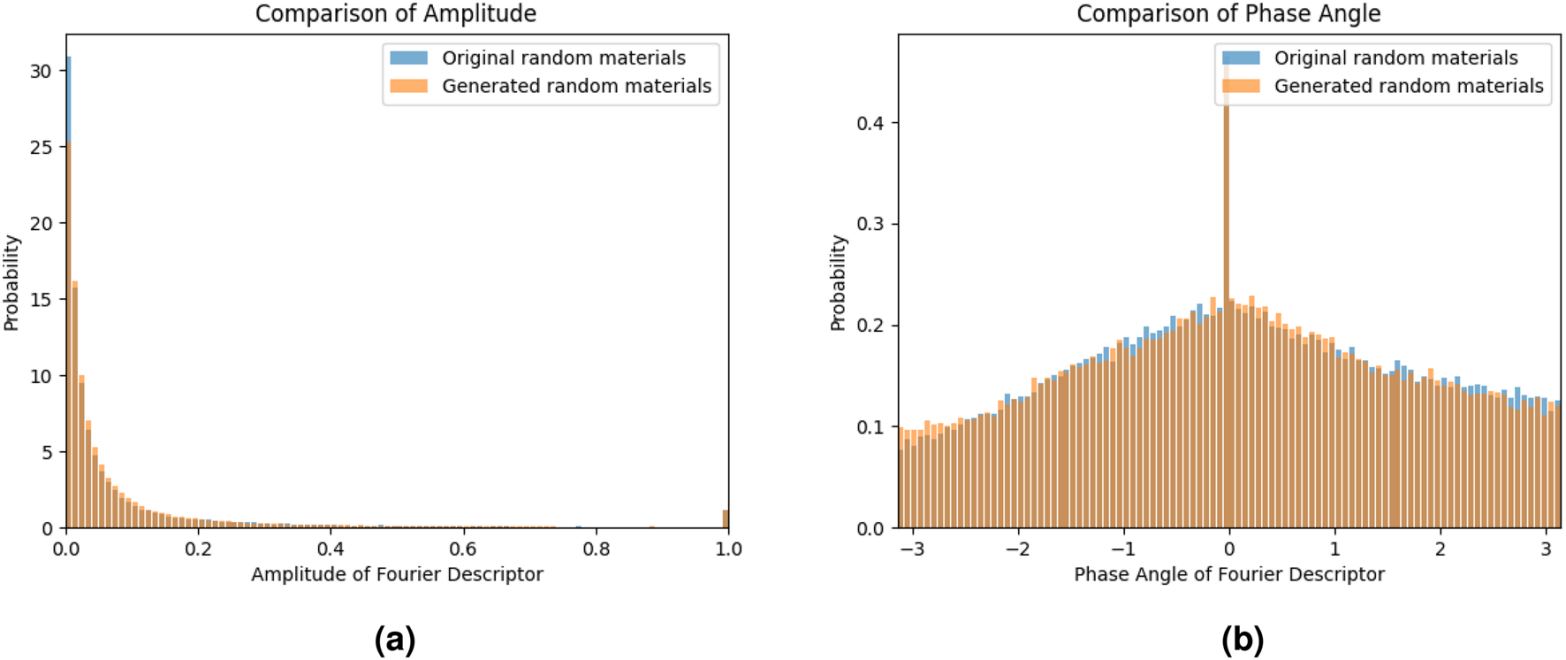
The feature distribution of Fourier descriptors for random microstructures; (**a**) The amplitude of Fourier descriptor. (**b**) The phase angle of Fourier descriptor.

This study undertakes a statistical examination by analyzing the histograms of the feature vectors derived from the Fourier descriptors of both original and reconstructed random inclusion materials, as illustrated in Fig.  8. The analysis reveals that the feature vectors corresponding to the reconstructed images’ Fourier descriptors exhibit a distribution trend closely mirroring that of the original images’ descriptors. Such consistency in distribution trends strongly suggests that the two sets of shapes of the phase boundary share similarities in visual and geometric characteristics, particularly highlighting parallels in their structure and composition.

### Randomness control and gradient material generation based on DDIM

As previously discussed, DDIM exhibits the capability to flexibly adjust the variance during sampling. Following the elimination of variance, the reverse process of the diffusion model transforms into a deterministic procedure. It allows for the exploitation of DDIM to comprehensively explore the latent space of the diffusion model and presents a promising avenue for controlling microstructure characteristics.

As a latent space model, the diffusion model encodes raw data into a latent space and subsequently utilizes a decoder to map the samples from the latent space back to the original data space. It establishes an equal-dimensional mapping between the latent space distribution and the original data distribution, where the noise distribution represents the latent space. Furthermore, since the diffusion model transforms discrete original image data into a continuous manifold structure, the mapping described above is inherently continuous as well. Building on this foundation, the diffusion model is capable of achieving the dynamic evolution of two microstructures by continuously interpolating between two noise samples. This capability not only allows for the control of microstructure randomness but also offers a quantifiable design space for the inverse design of random materials through distance measurements in latent space^17^.

It is worth noting that DDIM is sensitive to noise distribution. When linear interpolation is employed, $\lambda z_1+\left( 1-\lambda \right) z_2$ deviates from a normal distribution due to the superposition of normal distributions. Therefore, in this study, spherical interpolation was adopted^54,67^, which can be expressed as follows:

13 $$\begin{aligned} \begin{array}{l} {\textbf{x}}_{T}^{\left( \alpha \right) }=\frac{\sin \left( \left( 1-\alpha \right) \theta \right) }{\sin \left( \theta \right) }{\textbf{z}}_{T}^{\left( 0 \right) }+\frac{\sin \left( \alpha \theta \right) }{\sin \left( \theta \right) }{\textbf{z}}_{T}^{\left( 1 \right) }, \end{array} \end{aligned}$$

where $\theta =\arccos \left( \frac{\left( {\textbf{z}}_{T}^{\left( 0 \right) } \right) ^{\text {T}}{\textbf{z}}_{T}^{\left( 1 \right) }}{||{\textbf{z}}_{T}^{\left( 0 \right) }||{\textbf{z}}_{T}^{\left( 1 \right) }||} \right)$, and ${\textbf{z}}$ follows standard normal distribution.

**Figure 9:**
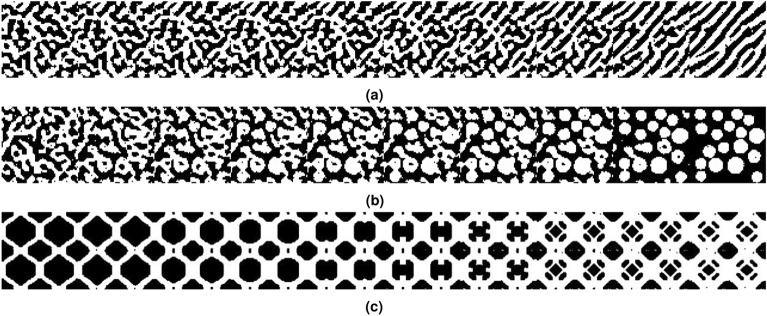
Randomness control and gradient materials based on DDIM.

Figure  9a,b illustrate the continuous interpolation decoding in the latent spaces of two microstructures, achieving dynamic evolution from random materials to texture materials and from random materials to circular inclusion materials, respectively. This method offers the potential to regulate the randomness within microstructures. Using the method outlined above, it also becomes possible to create gradient structures without the need for direct generation. Instead, gradient structures can be generated by combining intermediate structures from an extensive dynamic evolution process, as shown in Fig.  9c.

To achieve a nuanced quantification of randomness for various microstructures, this study leverages the energy of detail coefficients obtained via wavelet transform as a measure. The wavelet transform, distinguished by its ability to capture local features within images, such as edges, textures, and abrupt changes, is particularly adept at analyzing images characterized by non-stationary or abrupt features, thereby surpassing the capabilities of Fourier transform for this application. The mathematical formulation of the wavelet transform is given as follows:

14 $$\begin{aligned} W\left( a,b \right) =\int _{-\infty }^{\infty }{f}\left( t \right) \frac{1}{\sqrt{a}}\psi ^*\left( \frac{t-b}{a} \right) dt, \end{aligned}$$

where $f\left( t\right)$ is the original signal, $\psi \left( t \right)$ is the mother wavelet function, *a* is the scale parameter, *b* is the translation parameter, and * represents conjugation.

The discrete wavelet transform decomposes the image into approximate coefficients (capturing low-frequency components) and detail coefficients (encompassing high-frequency components), producing four sub-bands: LL (low-frequency, low-frequency), LH (low-frequency, high-frequency), HL (high-frequency, low-frequency), and HH (high-frequency, high-frequency). This decomposition allows for the extraction of diverse levels of information from an image, such as textures, edges, and local structures. Accordingly, the randomness index in this study is defined by the energy of the detail coefficients, estimated through the sum of squares formula:

15 $$\begin{aligned} E=\sum {|}LH|^2+\sum {|}HL|^2+\sum {|}HH|^2. \end{aligned}$$

**Figure 10:**
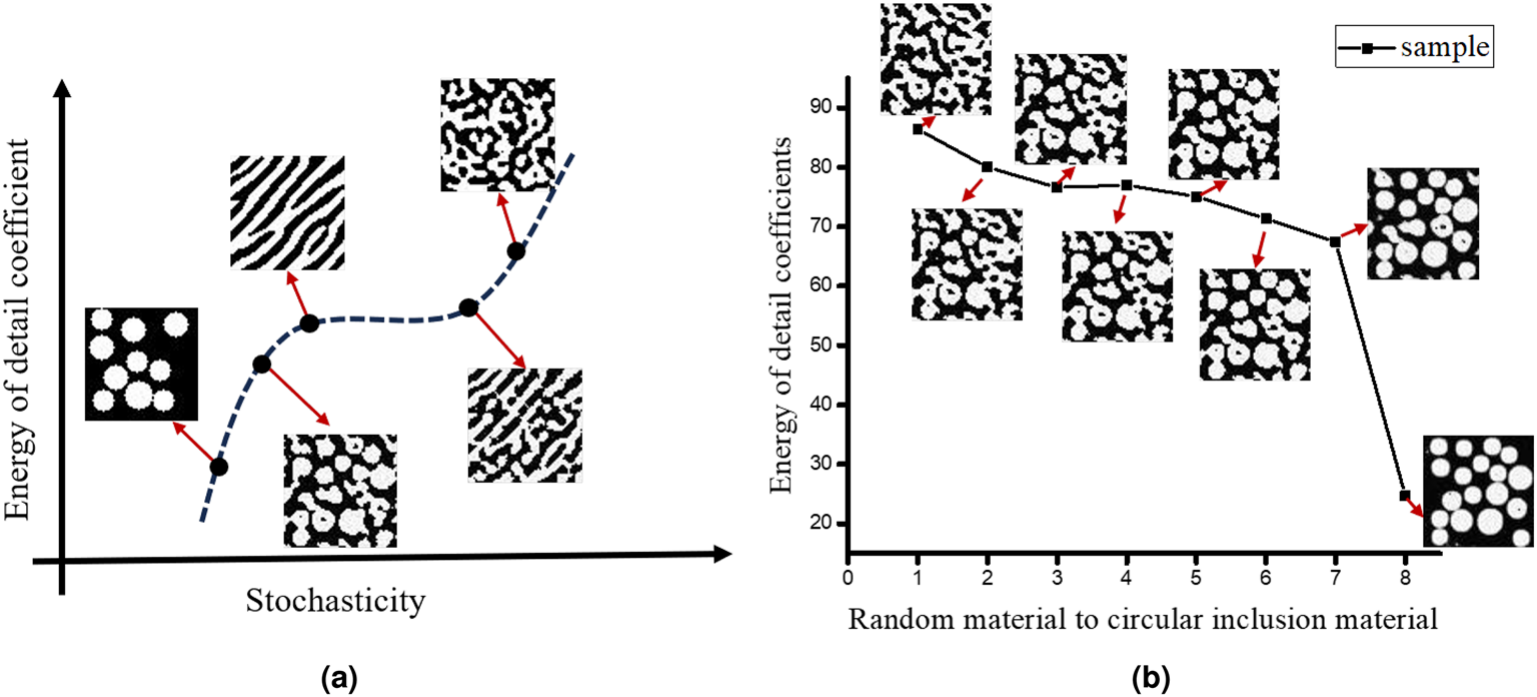
Randomness measurement of microstructure based on detail coefficient energy; (**a**) Comparison of randomness in different microstructures. (**b**) The evolution process of microstructures and corresponding randomness metric values.

A high energy level in the detail coefficients of a specific image region typically signifies the presence of irregular features, such as fine structures, textures, or edges. Conversely, a low energy level suggests a smoother and more uniform area. The characterization of randomness in circular inclusions, directional randomness, and quasi-random materials are effectively conducted using the energy of detail coefficients, with their respective energies approximated at 20, 80, and 90. The degree of randomness and energy magnitude in the evolution process from random materials to circular inclusions are shown in the Fig.  10. The results indicate that this indicator has good performance in measuring the randomness of irregular materials.

### Conditional generation of 3D random materials based on DDPM

**Figure 11:**
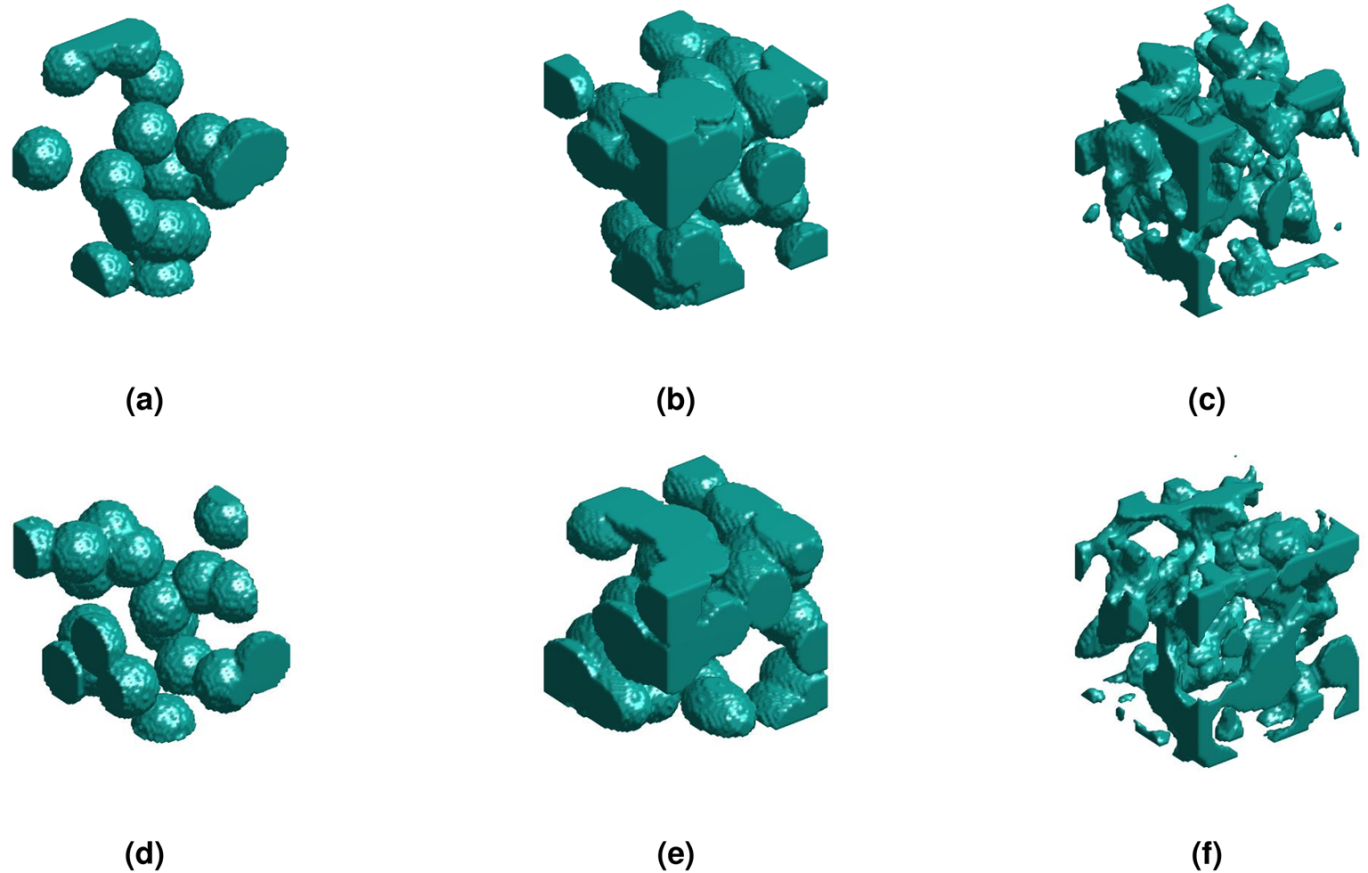
Comparison between original three-dimensional microstructure and reconstructed microstructure of composite materials; (**a**) Original spherical inclusion. (**b**) Original ellipsoidal inclusion. (**c**) Original random porous material. (**d**) Generated spherical inclusion. (**e**) Generated ellipsoidal inclusion. (**f**) Generated random porous material.

In microstructure reconstruction of composite, this study extends the diffusion model framework to 3D, and successfully achieves 3D reconstruction of spherical inclusion materials, ellipsoidal inclusion materials, and random materials, all with sizes of $64 \times 64 \times 64$. The three-dimensional microstructures of circular inclusions and ellipsoidal inclusion in the database were generated based on physical descriptors, while the random porous microstructures in database were generated based on random harmonic functions, with a sample size of 3000 each. The comparison between the original three-dimensional microstructures and the generated three-dimensional microstructures is shown in the Fig.  11.

**Figure 12:**
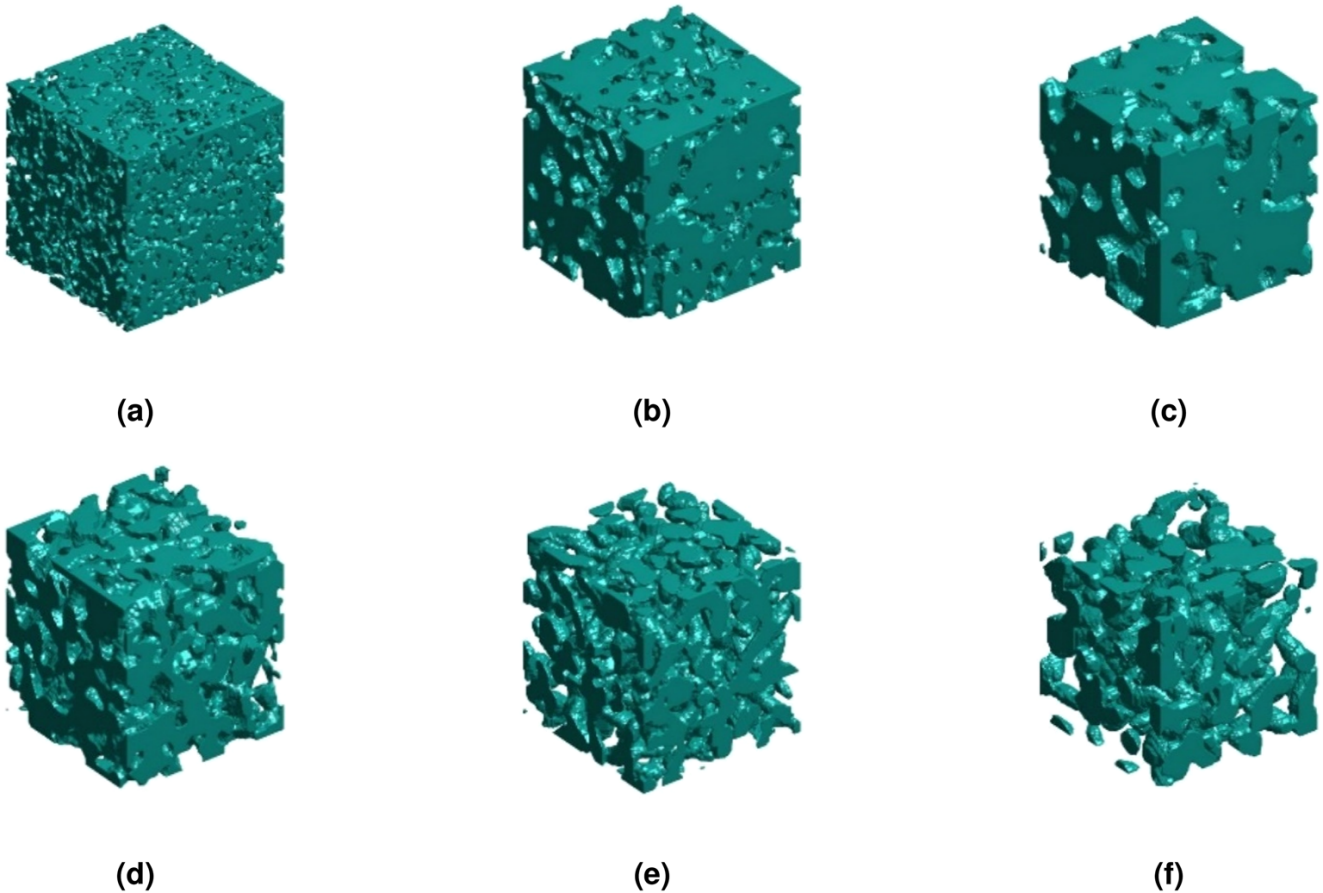
Three dimensional random porous materials generated by feature encoding embeddings.

Meanwhile, in this study, conditional generation was performed on three-dimensional random materials within a specific permeability range. The dataset required for three-dimensional conditional generation primarily sourced from publicly available research data in the literature^2^, which contains a massive database of 3D microstructures and various microstructure descriptors and labels. Finally, A total of 13,500 three-dimensional porous microstructures with permeability labels were used for training conditional diffusion models. Specifically, the permeability of three-dimensional materials was divided into six ranges as number labels, each of which serves as a feature encoding embedding combined with image and time encoding. The ranges of permeability are (0, 0.2), (0.2, 0.5), (0.5, 1.5), (1.5, 3.0), (3.0, 5.0), and (5.0, 5.0+), respectively. The generated results with the permeability from small to large in order of labels are shown in Fig.  12.

The verification of the permeability of random porous materials is carried out through the lattice Boltzmann numerical method,

16 $$\begin{aligned} f_i\left( {\textbf{x}}+{\textbf{c}}_i\delta t,t+\delta t \right) -f_i\left( \textbf{x,}t \right) =-\tau ^{-1}\cdot \left( f_i\left( \textbf{x,}t \right) -f_{i\left( eq \right) }\left( \textbf{x,}t \right) \right) , \end{aligned}$$

where *f* is the particle velocity distribution function, and the Boltzmann equation is essentially a conservative description of the spatiotemporal changes of *f*. ${\textbf{x}}$ and ${\textbf{c}}$ are the positions and velocities of particles, respectively. $\tau$ is the relaxation time. $f_{i\left( eq \right) }$ is the equilibrium distribution function. In this study, slip-free and rebound boundary conditions are applied at the two-phase interface. The initial fluid velocity is set to zero, and flow is induced by a constant pressure difference in the structure along the transport direction^68^. The initial condition entails a linear pressure gradient, and the relaxation time is maintained at 1.0.

Permeability can be determined using Darcy’s law, as follows:

17 $$\begin{aligned} {\bar{u}}=-\frac{\kappa \Delta p}{\mu d}. \end{aligned}$$

**Figure 13:**
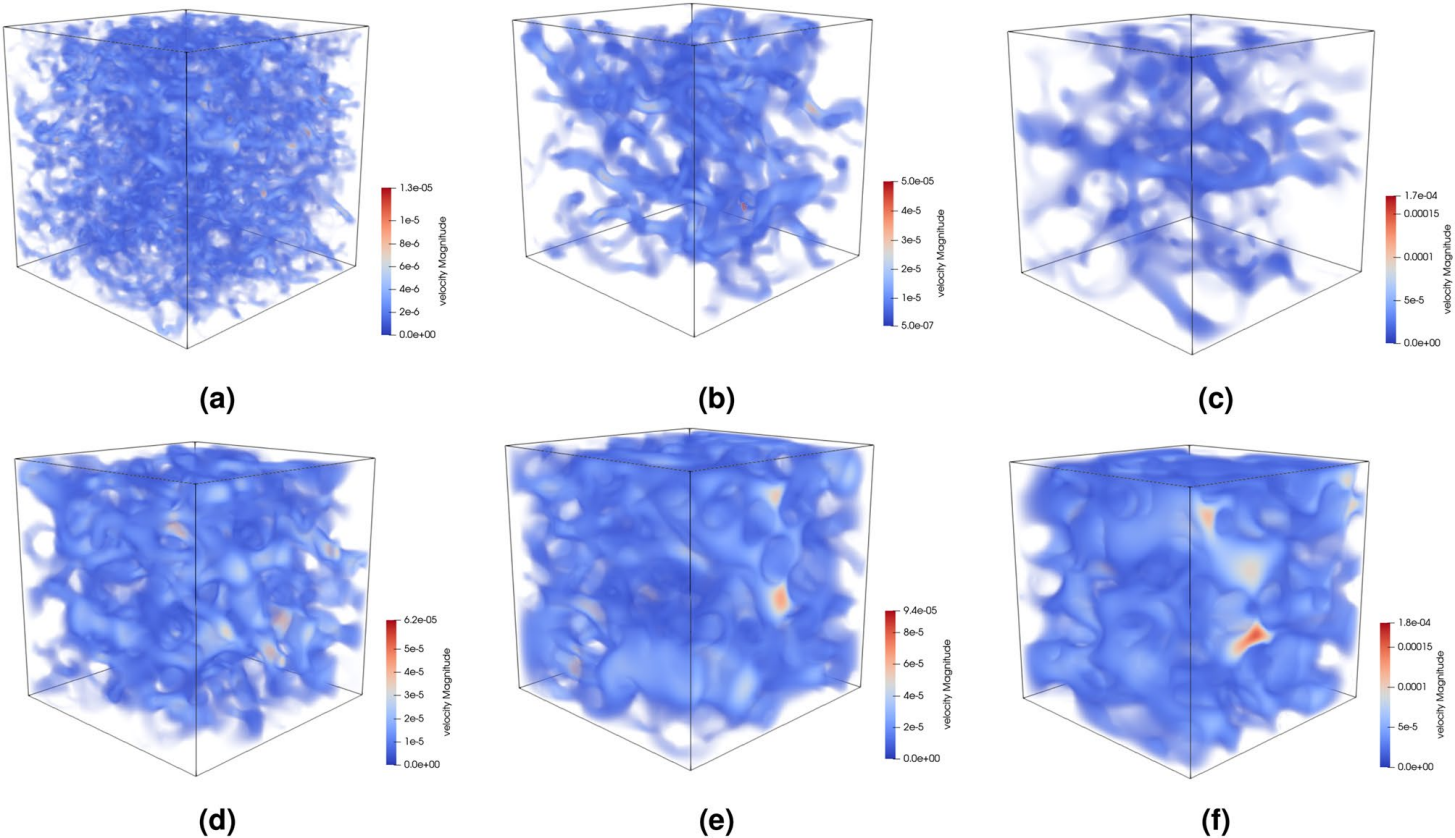
Velocity distribution in generated random porous material; (**a**) permeability = 0.14; (**b**) permeability = 0.44; (**c**) permeability = 0.66; (**d**) permeability = 1.83; (**e**) permeability = 4.57; (**f**) permeability = 10.93.

Among them, ${\bar{u}}$ is the average velocity, $\mu$ is the fluid dynamic viscosity, and $\varDelta p/d$ is the pressure gradient. Under the above conditions, the velocity distribution of the fluid in the pores inside the random porous material is shown in the Fig.  13. The permeability of these random materials is 0.14, 0.44, 0.66, 1.83, 4.57, and 10.93, respectively, which follows the range represented by feature encoding embedding.

## Conclusion

In this study, DDPM was successfully used to reconstruct the two-dimensional microstructure of various composite materials, including particle inclusion materials, quasi random materials, random materials, etc. In order to evaluate the quality of reconstruction, statistical comparisons were made using descriptors such as the two-point correlation function, lineal-path function, and Fourier descriptor, which quantitatively compared spatial relationships and boundary shapes. The results demonstrate the morphological similarity between the generated microstructure and the original microstructure, highlighting the diffusion model’s efficacy in microstructure reconstruction. Based on the above foundation, DDIM was also utilized for continuous interpolation in the latent space, enabling the regulation of microstructure randomness and the creation of gradient materials. This provides a new approach for the controllable application of randomness in material design.

Moreover, this study extended two-dimensional microstructure reconstruction to the three-dimensional framework and successfully reconstructed the three-dimensional microstructure of spherical inclusions, ellipsoidal inclusions, and random porous materials. Additionally, permeability was incorporated as the feature encoding embedding, allowing for the conditional generation of three-dimensional microstructures within a defined permeability range, providing a feasible approach for inverse design of three-dimensional random materials.

However, it’s important to acknowledge that this work is limited to defined permeability ranges in material inverse generation. Future research should explore precise permeability control and material design under multi-physical field coupling. Additionally, while diffusion models demonstrate excellence in microstructure reconstruction, they present challenges when used for forward optimization in material design because of their extensive latent spatial dimensions and unclear semantics. Combining the latent spatial dimensionality reduction operation of VAE and the excellent generation ability of diffusion models is also a direction that needs to be worked on in the future.

### Supplementary Information

Supplementary Information.

## Data Availability

Data associated with this research will be made available from the corresponding author on reasonable request.
