# Supplementary material for: Microstructure reconstruction of 2D/3D random materials via diffusion-based deep generative models
Source: Sci Rep. 2024 Feb 29;14:5041. doi: 10.1038/s41598-024-54861-9 (PMC10904791; doi:10.1038/s41598-024-54861-9)
Supplement: Supplementary file 1 — Supplementary Information. [file 41598_2024_54861_MOESM1_ESM.pdf]

Supplementary Methods

The database used in this study includes microstructures of various regular and random inclusions, including fiber inclusions, circular inclusions, texture microstructure, random inclusions, spinodal decomposition, Voronoi microstructure, fractal noise, and chessboard microstructure. Fiber inclusion materials and circular inclusion materials are reconstruction based on physical descriptors, including volume fraction, diameter (or length) of inclusions, etc. For fractal noise materials and Voronoi structured materials, the microstructure generation module in PoreSpy is used to create artistic images.

Among them, texture materials, spinodal decomposition materials and 2D/3D random materials are generated using random harmonic functions, and the specific expressions are as follows,

$$Z(x_1,x_2) = A(K_{1,i},K_{2,j}) \times [\cos(K_{1,i}x_1 + K_{2,j}x_2 + \phi_{ij}^{(1)}) + \cos(-K_{1,i}x_1 + K_{2,j}x_2 + \phi_{ij}^{(2)})]$$

where $\phi_{ij}^{(1)}$ and $\phi_{ij}^{(2)}$ $(i=1,2,...,N_1;\ j=1,2,...,N_2)$ are independent random variables that follow a uniform distribution within the $[0,2\pi]$; $K_{1,i},K_{2,j}$ represents the random wavenumber vector. k_1^L, k_1^U, k_2^L and k_2^U are respectively the boundaries of non-overlapping sub-domains. The amplitude can be expressed as,

$$A(K_{1,i},K_{2,j}) = \sqrt{4S_Z(K_{1,i},K_{2,j})(k_{1,i}^U - k_{1,i}^L)(k_{2,j}^U - k_{2,j}^L)},$$

where $\phi_{ij}^{(1)}$ and $\phi_{ij}^{(2)}$ $(i=1,2,...,N_1;\ j=1,2,...,N_2)$ are independent random variables that follow a uniform distribution within the $[0,2\pi]$; $K_{1,i},\ K_{2,j}$ represents the random wavenumber vector. k_1^L, k_1^U, k_2^L and k_2^U are respectively the boundaries of non-overlapping sub-domains. The amplitude can be expressed as,

$$A(K_{1,i},K_{2,j}) = \sqrt{4S_Z(K_{1,i},K_{2,j})(k_{1,i}^U - k_{1,i}^L)(k_{2,j}^U - k_{2,j}^L)},$$

where $S_Z(K_{1,i},K_{2,j})$ is the power spectral density function of the GRF.

The spectral density functions for texture and spinodal decomposition microstructures are,

$$S_Z(i,j) = \left(\tanh\left(20 - 100\sqrt{(K_{11}(i,j) - 0.5)^2 + (K_{22}(i,j) - 0.5)^2}\right) + 1\right)/2,$$

$$S_Z(i,j) = \left(\tanh\left(100 - 100\sqrt{(K_{11}(i,j))^2 + (K_{22}(i,j))^2}\right) + 1\right)/2.$$

The spectral density functions of two-dimensional and three-dimensional random microstructures are,

$$S_Z(i,j) = \left(c_1 \cdot c_2 \cdot \exp\left(-\left(\frac{c_1 \cdot K_{11}(i,j)}{2}\right)^2 - \left(\frac{c_2 \cdot K_{22}(i,j)}{2}\right)^2\right)\right)/4\pi,$$

$$S_Z(i,j,k) = \left(c_1 \cdot c_2 \cdot c_3 \cdot \exp\left(-\left(\frac{c_1 \cdot K_{11}(i,j,k)}{2}\right)^2 - \left(\frac{c_2 \cdot K_{22}(i,j,k)}{2}\right)^2 - \left(\frac{c_3 \cdot K_{33}(i,j,k)}{2}\right)^2\right)\right)/8\pi\sqrt{\pi},$$

c_i is the parameter related to the correlation length of the random field.
